# Molecular evidence of *Rdl* mutations linked to Fipronil resistance in urban populations of *Blattella germanica* in Iran

**DOI:** 10.1016/j.parepi.2026.e00530

**Published:** 2026-08-03

**Authors:** Shahin Saeedi, Hassan Akrami, Kourosh Azizi, Azim Paksa, Ahmad Gholami, Mitra Boroomand, Sahar Souri Pilangorgi, Ehsan Saki, Mozaffar Vahedi, Mehdi Miri, Aboozar Soltani

**Affiliations:** a Student Research Committee, Department of Biology and Control of Disease Vectors, School of Health, Shiraz University of Medical Sciences, Shiraz, Iran; b Gastroenterohepatology Research Center, Shiraz University of Medical Sciences, Shiraz, Iran; c Research Center for Health Sciences, Institute of Health, Department of Medical Entomology and Vector Control, School of Health, Shiraz University of Medical Sciences, Shiraz, Iran; d Department of Medical Entomology and Vector Control, School of Health, Shiraz University of Medical Sciences, Shiraz, Iran; e Biotechnology Research Center, School of Pharmacy, Shiraz University of Medical Sciences, Shiraz, Iran; f Department of Biostatistics, School of Medicine, Shiraz University of Medical Sciences, Shiraz, Iran

**Keywords:** Urban pest, German cockroach, *Blattella germanica*, Fipronil, Insecticide resistance, *Rdl*

## Abstract

**Background:**

The German cockroach is a major urban pest and a persistent public health threat, largely due to its ability to rapidly develop resistance to commonly used insecticides. Fipronil, a phenylpyrazole insecticide widely incorporated into bait formulations, is increasingly compromised by resistance in field populations. This study evaluated the susceptibility of four urban *B. germanica* populations from Iran to fipronil and investigated the presence of Rdl gene mutations associated with phenylpyrazole resistance.

**Results:**

Topical bioassays revealed substantial variation in susceptibility, with resistance ratios ranging from 1.77 to 13.35 across populations, indicating heterogeneous exposure histories and potential genetic differentiation. Sequencing of a 245-bp fragment of the Rdl gene revealed no occurrences of the well-known A302S mutation; however, the A299S and A301S mutations, previously linked to fipronil resistance, was detected in two populations exhibiting the highest levels of resistance. Haplotype reconstruction of 21 individuals identified 17 haplotypes, suggesting considerable genetic diversity that may reflect multiple evolutionary origins of Rdl variants and ongoing selective pressure. However, larger sample sizes would be required to confirm this pattern.

**Conclusion:**

Field populations of *Blattella germanica* exhibited variable susceptibility to fipronil, ranging from susceptible to moderately resistant. Molecular analysis did not detect any established target-site resistance mutations in A302S, although A299S and A310S substitution were identified in two populations and require further investigation. These findings suggest that fipronil remains an effective control agent in many urban environments; however, reduced susceptibility in some populations underscores the importance of ongoing resistance monitoring and integrated pest management strategies to mitigate future resistance development.

## 1 Introduction

The German cockroach (Jones, Goven, Froger, Bantz, & Raymond) is a prevalent urban pest and poses substantial indoor public health risks (Vargo et al., 2021). Cockroaches contribute to the spread of respiratory, fungal, and bacterial diseases through their faeces, body fragments, and shed exoskeletons (Brenner and Kramer, 2019). Infestations can lead to significant economic impacts, including food contamination, increased medical expenses due to allergen exposure, and expenses related to regulatory compliance (Hassan Nasirian, 2019; Wang et al., 2021).

Effective management of cockroach populations contributes substantially to the mitigation of related public health risks (McPherson et al., 2021). Chemical interventions remain the primary and most effective strategy for controlling German cockroach populations (Brenner and Kramer, 2019). Pest management professionals (PMPs) rely heavily on residual insecticide sprays and baits, which constitute the main control tools for these vectors (C.-Y. Lee and Wang, 2021b).

The German cockroach reproduces rapidly, producing three to four generations per year, which accelerates adaptive responses to insecticidal pressure (Rust et al., 1995). Nevertheless, extensive and prolonged use of insecticides, such as pyrethroids, neonicotinoids, essential oils, and indoxacarb, has been employed to control German cockroaches (González-Morales et al., 2022) (Hu et al., 2020; C.-Y. Lee and Wang, 2021a; Phillips et al., 2010; Scharf and Gondhalekar, 2021). As a consequence, field populations have developed resistance across multiple chemical groups-organochlorines, organophosphates, carbamates, and pyrethroids-contributing to observed control failures (Enayati and Motevali, 2007; Kakeh-Khani et al., 2020; Rust et al., 1995; Zhu et al., 2016).

Fipronil, a relatively new insecticide, has been commonly used for three decades to control German cockroach infestations (Castilhos et al., 2019; Kaakeh et al., 1997; Singh et al., 2021). As a phenylpyrazole insecticide, fipronil disrupts the nervous system and behavior of insects by blocking GABA- and glutamate-gated chloride channels, leading to uncontrolled neural excitation and eventual death (D. Buckingham et al., 2017). Over the past decades, there has been a significant increase in global reports of fipronil resistance among *B. germanica* populations (González-Morales et al., 2022; Kassiri et al., 2020; Khoobdel et al., 2022b; H Nasirian et al., 2006a; Wu and Appel, 2017).

Rdl gene (resistance to dieldrin) mutations have been repeatedly associated with target-site resistance to cyclodiene and phenylpyrazole insecticides in various pest species (Liu et al., 2020).

In the German cockroach, the most widely reported substitution is A302S, which confers high resistance to dieldrin and fipronil. Interestingly, analogous amino-acid changes at nearby positions such as A296S, A299S and A301S have been identified in other insect taxa, including *Anopheles* mosquitoes, *Musca domestica, Nilaparvata lugens*, and *D. melanogaster* (Garrood et al., 2017; Liu et al., 2020; Ozoe et al., 2015; Remnant et al., 2014), where they have also been implicated in GABA receptor-mediated resistance.

Given the limited data on *Rdl* variation in *B. germanica*, this study investigates the genetic diversity and haplotype structure of the *Rdl* gene in populations collected from different urban environments and reports a previously uncharacterized substitution, A299S and A301S. This resistance is largely attributed to mutations in the Rdl gene, which encodes a subunit of the GABA-gated chloride channel, thereby reducing target-site sensitivity to fipronil (Ang et al., 2013; González-Morales et al., 2022; Li et al., 2021). These findings underscore the importance of ongoing molecular surveillance of Rdl mutations in cockroach populations to inform evidence-based resistance management programs.

## 2 Material and methods

### 2.1 Collection and Rearing

Between 2023 and 2024, four field populations of *B. germanica* were collected from public and residential settings across two Iranian cities: a university dormitory in Shiraz (29°38′35.44″N, 52°30′44.57″E), a residential apartment in Shiraz (29°37′19.19″N, 52°32′27.14″E), a fast-food restaurant in Tehran (35°42′46.83″N, 51°24′24.06″E), and Namazi Hospital in Shiraz (29°35′28.38″N, 52°35′01.06″E). All four field populations were sourced from locations with documented histories of insecticide application. A laboratory-susceptible strain, maintained under insecticide-free conditions for over 30 years at the insectary of the Department of Vector Biology and Control of Disease, School of Public Health, Tehran University of Medical Sciences, was used as the untreated control throughout all bioassays (Ladonni, 2001).

The cockroaches were reared separately in standard containers under controlled environmental conditions (24 ± 2 °C, 30–50% relative humidity, and a 12:12 L:D photoperiod). They were provided with food (dog food), water, and cardboard harborages as shelter (Gemeno Marín et al., 2011).

### 2.2 Insecticide and bioassay

Technical grade of fipronil, with a purity of 95%, was obtained from Moshkfam-Fars™ Chemical Company, Shiraz, Iran. The 50%. 90%,95% and 99% lethal dose (LD_50,_ LD_90,_ LD_95_ and LD_99_) of each population was determined using topical application bioassay (Nasirian et al., 2006b). Groups of 20 adult male cockroaches were briefly anesthetized with CO_2_ in a plastic Petri dish. Fipronil was topically applied in acetone using a micro-applicator (Hamilton, Reno, NV) equipped with a 50 μl glass syringe (Hamilton Co.), delivering 1 μl onto the ventral thorax of each cockroach (Scott et al., 1990). Serial dilutions of fipronil were prepared in acetone to achieve final doses ranging from 0.0002 to 5.685 ng/insect, corresponding to concentrations of 0.001, 0.01, 1, 2.5, 5, 7, 10, 15, and 25 ppm. For each of the five strains, bioassays were conducted using nine concentrations, with three independent replicates per concentration (540 individuals per population). Mortality was assessed at 24-, 48-, 72-, and 96-h post-treatment by gently probing each individual with forceps. Cockroaches that failed to exhibit coordinated locomotion in response to mechanical stimulation were recorded as dead. Final mortality rates were determined at 96 h post-treatment (González-Morales et al., 2022).

### 2.3 DNA extraction and Rdl gene sequencing

To investigate target-site resistance mechanisms, five adult males from each of the four field populations of *B. germanica* and one individual from the laboratory-susceptible strain (21 samples) were screened for Rdl gene mutations associated with the A302S amino acid substitution (Ang et al., 2013). The heads of individual cockroaches were homogenized for 30 s, and DNA was extracted using the Smbio™ DNA extraction Kit according to manufacturer's instructions. A 245-bp genomic fragment of the GABA receptor gene that includes the *Rdl* mutation site was amplified with the primers BG-Rdl-F (5′-GTGCGGTCCATGGGATACTA-3′) and BG-Rdl-R (5′-AACGACGCGAAGACCATAAC-3′) (M. Kristensen et al., 2005). A negative control with no template DNA was included in every PCR run. The following thermal cycle program was used for amplification: Initial activation at 95 °C for 5 min followed by 40 cycles of 94 °C for 30 s, 60.0 °C for 30 s, and 72 °C for 30 s and a final extension at 72 °C for 5 min (Gondhalekar and Scharf, 2012). Two microliters of each PCR product were electrophoresed on a 1.2% agarose gel to confirm the presence of bands with sized estimated. The remaining PCR product was sequenced by Sanger sequencing method. Each sequence was manually inspected for the GCC to TCC, TCC to AGC and GCA to TCA mutation that results in the A302S, A301S and A299S substitutions**.**

### 2.4 Haplotype reconstruction and confirmation

Genotype data quality was evaluated using Chromas v2.6.6 within BioEdit version 7.2.5 and MEGA 11 (Hall, 1999; Kumar et al., 2018). Rdl gene sequences obtained from 21 specimens, comprising 20 field-collected individuals and one susceptible strain from four populations, were aligned and analyzed in DnaSP v6 for haplotype identification and reconstruction (Librado and Rozas, 2009). Genetic relationships among haplotypes were visualized by constructing a median-joining haplotype network in PopART v1.7 (Leigh, Bryant, Nakagawa, and Evolution, 2015). Haplotype assignments were further confirmed through manual examination of sequence alignments and validation of polymorphic nucleotide sites.

### 2.5 Mutation analysis

For molecular analysis, DNA sequences were aligned and analyzed using MEGA11 and BioEdit version 7.2.5 software to identify point mutations, focusing on the A302S/N substitution associated with fipronil resistance (Ang et al., 2013). Sequence comparisons between field-collected and susceptible *B. germanica* strain were conducted to evaluate mutation frequencies and patterns. The reference sequence used was the complete coding sequence of the *B. germanica* GABA-gated chloride channel, deposited in GenBank under accession number MW267921.1 (Jones et al., 2021).

### 2.6 Statistical analysis of bioassay data

The LD_50_, LD_90_, LD_95,_ and LD_99_ values for each cockroach population were determined using log-dose probit-mortality analysis. Probit analysis was done using R software version 4.3.2 with the STATS and ggplot2 package. When necessary, Abbott's formula correction was applied for data of mortality (Abbott, 1925). The lethal dose ratio at LD_50_ was then used to calculate resistance ratios (Valles et al., 1997). To compare mortality at each fipronil concentration with the acetone control within each population, a chi-square test of independence was performed. For each concentration, a 2 × 2 contingency table was constructed (control vs. treatment, dead vs. alive). The resulting *p*-values were adjusted using the Bonferroni correction. A corrected p-value <0.05 was considered statistically significant.

In addition, to confirm the dose-dependent relationship independently of the probit model, Spearman correlation analyses were conducted between log-transformed dose and mortality proportion for each population (using only concentrations >0 ppm).

Goodness-of-fit of the probit models was assessed using the Pearson chi-square statistic. A non-significant p-value (*p* > 0.05) indicates that the model adequately describes the observed dose-mortality relationship.

Resistance ratios (RRs) and resistance-level classifications were adopted from previous studies on German cockroach insecticide resistance and other urban insect pests. Populations with RR ≤ 1 were considered susceptible, those with RR > 2 as exhibiting low resistance, RR > 5 as moderate resistance, and R*R* > 10 as high resistance (R. Y. Chai and Lee, 2010a; González-Morales et al., 2022; K.-M. Lee and Lee, 2002; Öz et al., 2021).

## 3 Results

### 3.1 Bioassay findings

**Fig. 1:**
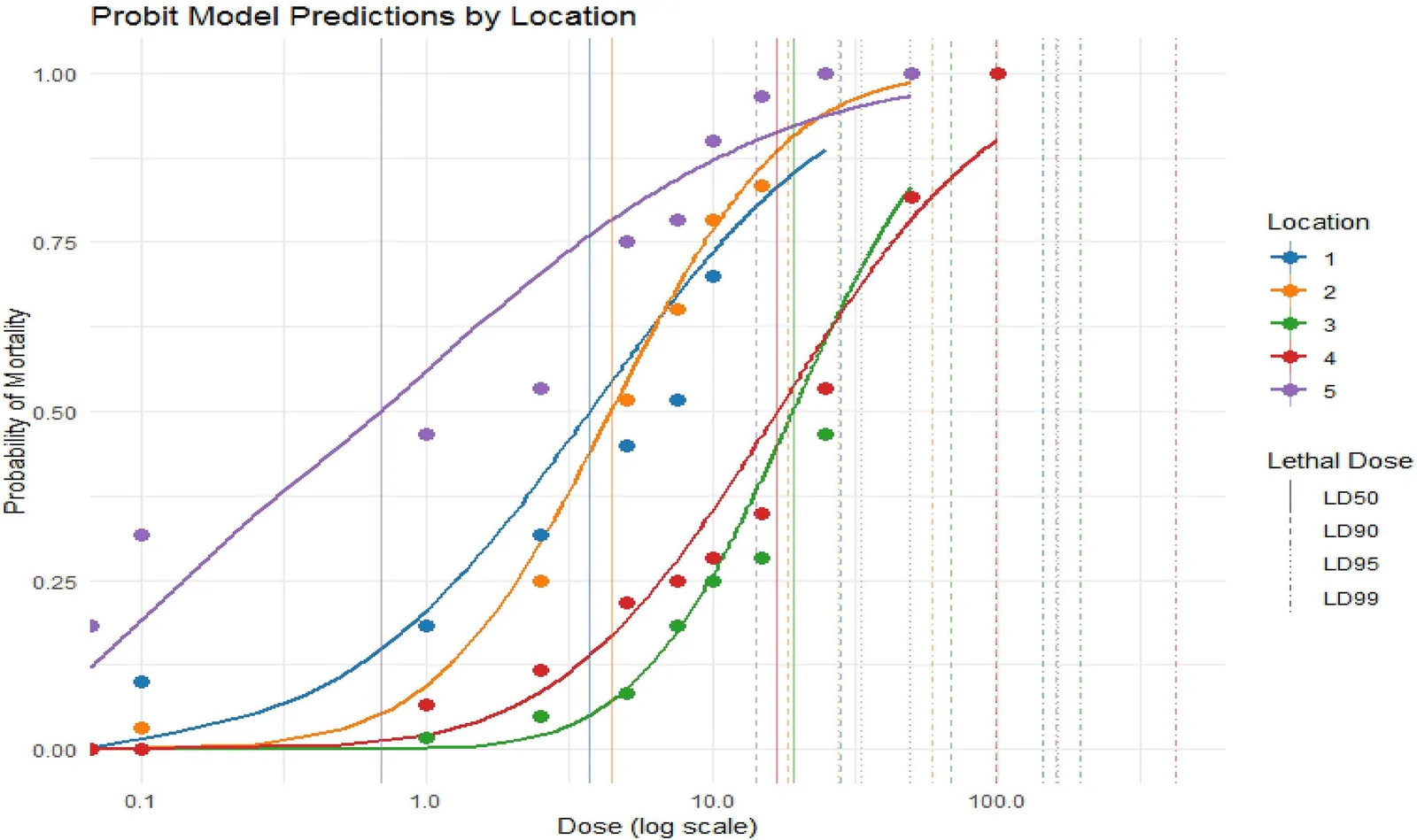
Probit model predicts mortality in [location 1: Dormitory, Location 2: Apartment, Location 3: Hospital, Location 4: Fast-food restaurant and Location 5: laboratory standard strain]. LD_50_ (solid), LD_90_ (dashed), LD_95_ (dash-dot), LD_99_ (dotted).

The dose–response relationship of the *B. germanica* populations collected from four different locations was evaluated using probit regression. In all locations, there was a statistically significant positive association between the log-transformed dose and mortality (*p* < 0.001), indicating that the probability of death increased with increasing doses of the insecticide (Fig. 1). The estimated coefficients of the probit models are presented in Table 1**.** The Pearson chi-square goodness-of-fit test yielded non-significant *P*-value for all populations (*P* > 0.05, ranging 0.0598 to 0.392) indicates that the model adequately describes the observed dose-mortality relationship **(**Table 1**)**. The intercept values differed among locations, suggesting variability in baseline susceptibility, while the slopes were all positive and significant, reflecting a consistent dose-dependent increase in mortality.

**Table 1:** Probit analysis of mortality (at 96 h after treatment) of four *B. germanica* populations and a susceptible strain treated with fipronil.

Strains	Estimate		St. Error	P-value	95% confidence intervals	P-value for Pearson chi-square goodness-of-fit statistics
Dormitory	(Intercept)	−0.11	0.06	<0.0001	−0.247, 0.010	0.392
	log dose	0.49	0.03		0.428, 0.557	
Apartment	(Intercept)	−0.59	0.08	<0.0001	−0.766, −0.443	0.0843
	log dose	0.55	0.04		0.475, 0.636	
Hospital	(Intercept)	−2.02	0.20	<0.0001	−2.417, −1.667	0.121
	log dose	0.91	0.08		0.751, 1.093	
Fast-food restaurant	(Intercept)	−2.30	0.22	<0.0001	−2.745, −1.901	0.176
	log dose	1.07	0.10		0.889, 1.274	
Susceptible strain	(Intercept)	0.75	0.10	<0.0001	0.607, 0.916	0.0598
	log dose	0.55	0.03		0.481, 0.627	

The probit models were used to calculate the lethal doses for 50%, 90%, 95% and 99% mortality (LD_50_, LD_90_, LD_95_ and LD_99_) for each population (Table 2).

**Table 2:** Fipronil dose-response results in the susceptible strain and four *B. germanica* populations recently collected from (Hospital, Apartment, Dormitory and Fast-food Restaurant).

Strains	N	LD_50_ (50% CL)	LD_90_ (90% CL)	LD_95_ (95% CL)	LD_99_ (99% CL)	Resistance ratio based on LD_50_	Resistance Status (Standard Criteria)
Dormitory	540	1.21	17.17	36.03	144.32	1.77	R > 1/low resistance
Apartment	540	2.91	30.04	58.02	169.28	4.27	*R* < 5/ moderate resistance
Hospital	540	9.08	36.91	54.88	115.46	13.35	R > 10/ high resistance
Fast-food restaurant	540	8.49	28.10	39.42	74.35	12.48	R > 10/ high resistance
Susceptible Strain	180	0.68	2.53	4.93	17.08	–	–

Susceptible populations had the lowest LD_50_, LD_90_, LD_95_ and LD_99_ values, indicating the greatest insecticide sensitivity. Hospital and Fast-food restaurant strains showed the highest resistance ratios (12.48 and 13.35), indicating relative resistance, while dormitory and apartment populations exhibited intermediate susceptibility with resistance ratios of 1.77 and 3.36, respectively. These findings demonstrate substantial variation in insecticide sensitivity across populations from different locations. Such differences may reflect prior exposure to insecticides, environmental conditions, or genetic variation among populations.

#### 3.1.1 Comparison of mortality between treatment groups and control

To determine whether fipronil concentrations caused significantly higher mortality than the control, a chi-square test of independence was performed for each concentration within each population. In the susceptible strain, all tested concentrations (0.001–25 ppm) resulted in significantly higher mortality than the control (all *p* < 0.001). In the low-resistant Dormitory strain, the lowest concentration (0.001 ppm) already showed a significant difference (χ^2^ = 9.730, df = 1, *p* = 0.0018), and all higher concentrations were also significant (p < 0.001).

In the high resistant Apartment strain the concentration of 0.001 ppm did not differ significantly from the control (χ^2^ = 2.034, df = 1, *p* = 0.1538), but concentrations ≥0.1 ppm were all significantly different (*p* ≤ 0.0064). In the highly resistant Hospital strain, no mortality was observed at 0.001 and 0.1 ppm, and the first significant difference appeared at 1 ppm (χ^2^ = 5.217, df = 1, *p* = 0.0224). In the Fast-food restaurant strain, also highly resistant, concentrations of 0.001 and 0.1 ppm yielded no mortality, and even at 1 ppm the difference was not significant (χ^2^ = 3.077, df = 1, *p* = 0.0794); significant differences started at 2.5 ppm (χ^2^ = 7.434, df = 1, *p* = 0.0064). These chi-square results are fully consistent with the resistance ratios derived from probit analysis, confirming that higher fipronil resistance is associated with a higher threshold dose required to achieve a significant increase in mortality over the control.

#### 3.1.2 Correlation analysis between dose and mortality

In addition to probit regression, Spearman correlation analyses were performed between log-transformed dose and mortality proportion for each population (using concentrations >0 ppm). Positive correlations were observed in all populations. Spearman correlation coefficients ranged from 0.815 (Hospital strain, *p* = 0.0074) to 0.979 (susceptible strain, p < 0.001). These results independently confirm a dose-dependent increase in fipronil-induced mortality, supporting the validity of the probit model.

### 3.2 Mutation finding

**Fig. 2:**
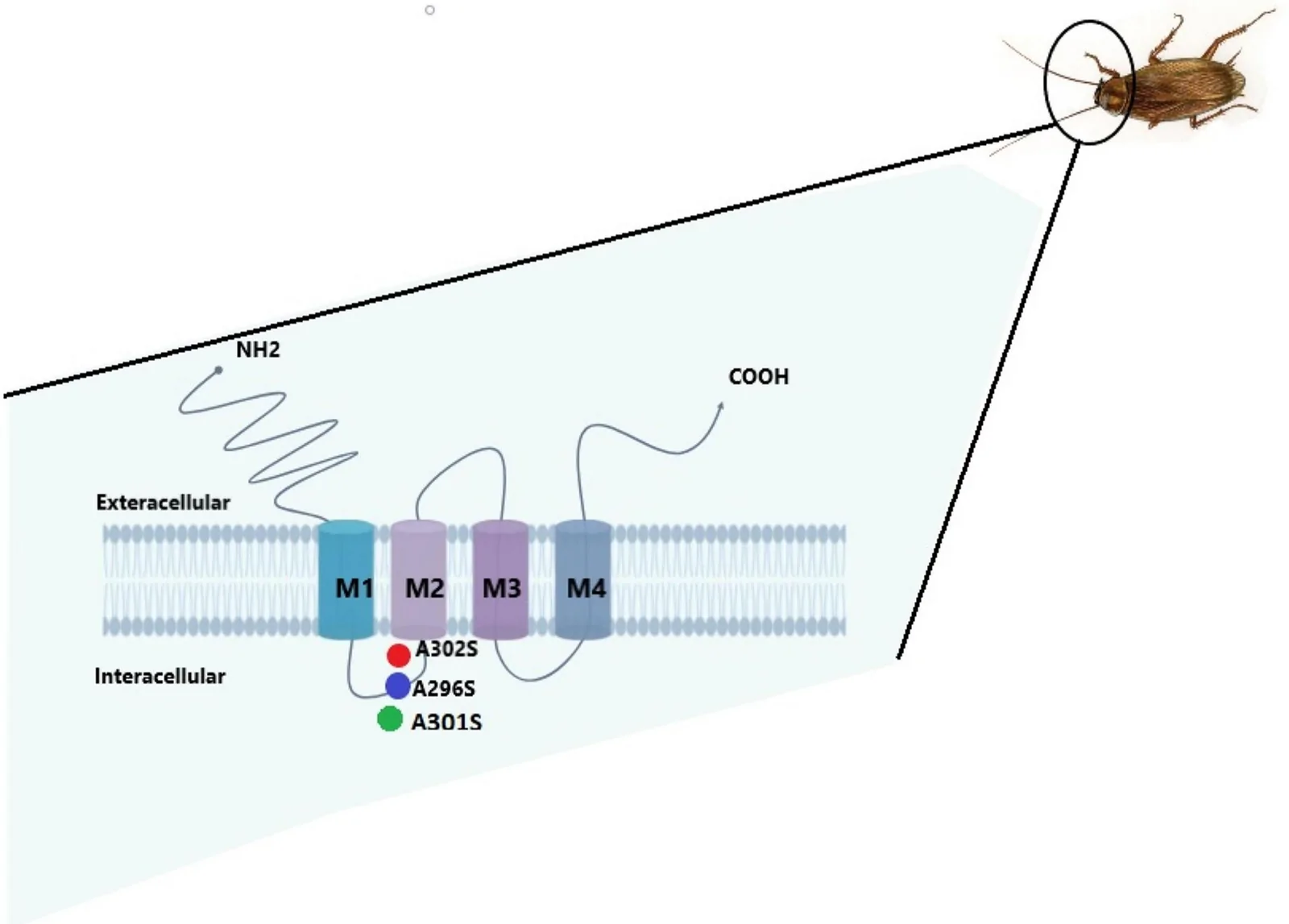
Schematic diagram of polymorphic sites in the Rdl monomer. The phenylpyrazole resistance-associated substitution A299S is indicated by a blue dot, the A301S substitution by a green dot, and the well-characterized A302S resistance-associated substitution by a red dot. (For interpretation of the references to colour in this figure legend, the reader is referred to the web version of this article.)

**Fig. 3:**
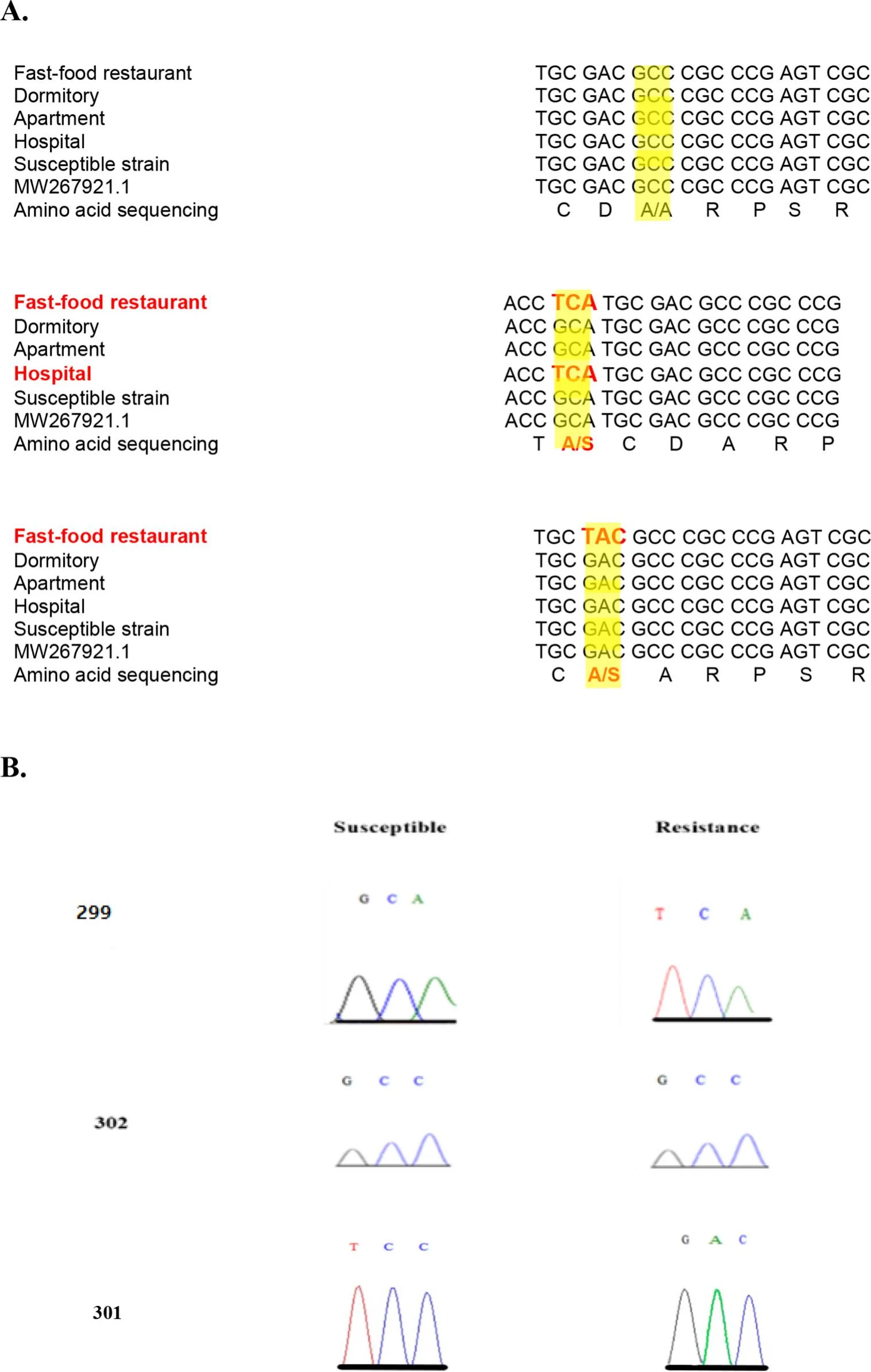
A: Nucleotide sequences of the TM2 region of the *B. germanica* Rdl gene, which includes the point mutation that results in the A302S, A301S and A299S substitution. Representative sequences from four field-collected German cockroach populations were aligned against the reference sequence (MW267921.1), with the A299S A302S and A301S, regions highlighted. B. Direct sequencing chromatograph showing two individual genotypes of the phenyl pyrazole insecticide resistance-related sites.

We analyzed a 245-bp fragment of the *Rdl* (GABA receptor) gene encompassing the A302S, A301S and A299S mutation sites previously associated with phenylpyrazole (fipronil) resistance (Fig. 2). Samples included a susceptible strain and four field-collected *B. germanica* populations (*n* = 5 per population). Based on earlier reports linking fipronil resistance to the A301S and A299S substitutions, we screened these loci to assess their occurrence in the studied populations. The A302S mutation was not detected in any of the field populations, whereas the A299S mutation was identified in individuals from the Fast-food restaurant and hospital populations (Fig. 3**,** Table 3).

**Table 3:** Frequencies of Rdl genotypes in different field populations of *B. germanica.*

Populations	n	A302S (S/S)	A299S (A/S)	A301S (A/S)
Hospital	5	5	1	0
Fast-food restaurant	5	5	1	1
Apartment	5	5	0	0
Dormitory	5	5	0	0
Susceptible strain	1	1	0	0

### 3.3 Haplotype network finding

Sequencing of a 245-bp fragment of the Rdl gene from 21 *B. germanica* individuals revealed 17 unique haplotypes, indicating exceptionally high genetic diversity (Hd = 1.000 ± 0.020) and substantial nucleotide diversity (π = 0.0405 ± 0.007), with 24 polymorphic sites and 27 mutations detected.

Haplotype network analysis identified 18 haplotypes across populations collected from dormitories, apartments, hospitals, fast-food restaurants, and a laboratory strain. Hap_18 (accession MW267921.1, USA) was positioned at the center of the network, suggesting it represents a common ancestral haplotype. Populations from hospitals and fast-food restaurants exhibited greater mutational distances and higher genetic divergence, whereas the laboratory strain showed reduced diversity and clustered closely around the central haplotype.

**Fig. 4:**
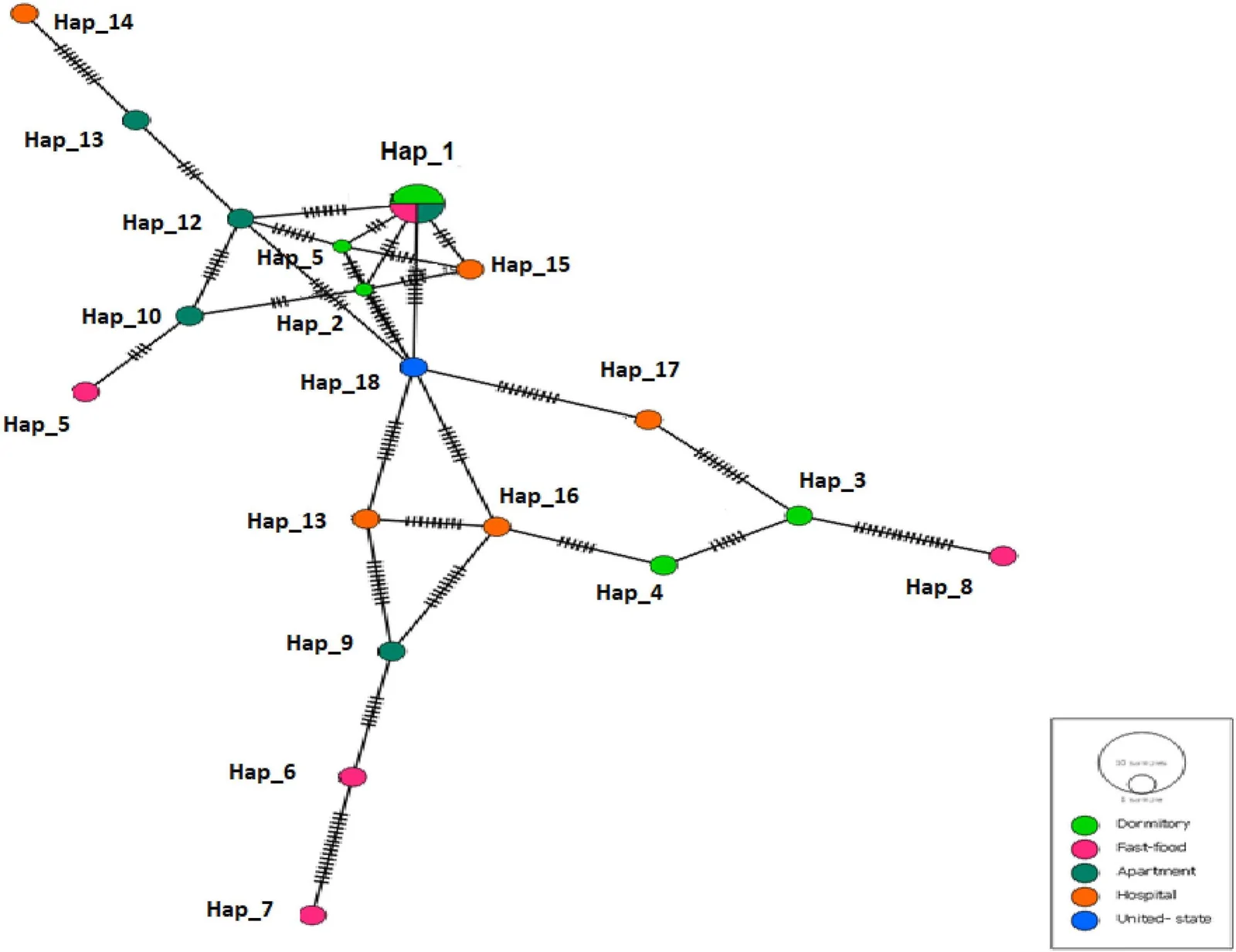
Genealogy of Rdl haplotypes is represented as a network. Circle size corresponds to haplotype frequency across populations.

Two haplotypes (H1 and H13) carried the G → T substitution at position A299S and A → S in position 301, previously linked to phenylpyrazole insecticide resistance. Tajima's D was negative (D = −0.88357) but not statistically significant (*p* > 0.1), and the network displayed a star-shaped pattern (Fig. 4).

## 4 Discussion

This study reveals significant variation in susceptibility and resistance to fipronil among German cockroach (*Blattella germanica*) populations. Using probit analysis across a concentration range of 0.001 to 25 μl in 1000 μl acetone, the susceptible strain exhibited an LD₅₀ of approximately 0.68 ppm, while resistant field populations showed LD₅₀ values more than 20-fold higher, indicating substantial resistance ratios (Scharf & Gondhalekar) **(**Table 4**).** These findings align with previous reports documenting highly variable mortality rates (8.5–98.4%) in field populations exposed to fipronil baits after 7 days (Khoobdel et al., 2022a; H Nasirian, 2007; H Nasirian et al., 2006b). Probit analysis effectively described the dose-response relationships, providing reliable mortality predictions based on the cumulative normal distribution a standard approach for evaluating insecticide efficacy in pest species (González-Morales et al., 2022) **(**Table 4**).**

**Table 4:** Summary of field studies quantifying fipronil resistance in German Cockroach (*Blattella germanica*) populations.

Time period	Location	RR^a^ range	Type of treatment	Assay used	Time of mortality assessment (Castilhos et al.)	References	Notes
1997–2010	United States	1.0–1.8 (1.8–7.7) b	Topical	Dose-response	4	Scott and Wen (1997)	Baseline susceptibility
	United States	1.0–1.3	Topical	Dose-response	1	Valles et al. (1997)	Low resistance
	United States	1.2–>17^c^, ^d^, ^e^	Topical	Dose-response	3	Holbrook et al. (2003)	Emerging high resistance
	United States	8.7–9.3	Topical	Dose-response	3	Wang et al. (2004)	Moderate resistance
	Denmark	1–15	Topical	Dose-response	3	Kristensen et al. (2005)	Variable resistance
	Iran	1–2.6	Topical	Dose-response	3	Nasirian et al. (2006a)	Low resistance; permethrin cross-check
	Singapore	1.0–10.0	Topical	Dose-response	2	Chai and Lee (2010a)	Moderate resistance
2011–2020	United States	37.9	Topical	Dose-response	3	Gondhalekar and Scharf (2012)	High resistance
	Singapore	1.2–3.0 (10.8–25.8) b	Topical	Dose-response	2	Ang et al. (2013)	Increasing secondary kill resistance
	Puerto Rico	5.6 (15.9)	Topical	Dose-response	2	Ko et al., (2016)	Moderate resistance
	United States	0.9–1.4 (2.5–25.0) b	Topical	Dose-response	5	Liang et al., (2017)	Variable, high secondary
	United States	2.0–8.7	Topical	Dose-response	3	Wu and Appel (2017)	Moderate resistance
	United States	6–23	Topical	Dose-response	2	DeVries et al. (2019)	High resistance
	Taiwan	1.5–3.8	Surface contact	Time-course (LT_50_)	7	Hu et al. (2020)	Low-moderate; LT_50_ focus
2021–2025	United States	∼27.7^f^	Ingestion	Discriminating doses	3	Lee et al. (2022)	Stable high resistance
	United States	22.4–37.2	Topical	Dose-response	4	González-Morales et al. (2022)	Linked to A302S mutation
	Indonesia	12.21	Topical	Dose-response	2	)Van Dini et al., (2023)	Moderate-high resistance
	Iran	N/A (76.5% decline at 7 days)	Bait (gel/powder)	Field trial (sticky traps)	7	Khoobdel et al. (2022a)	Efficacy study; no RRa, high mortality with 0.02% fipronil; potential A302S link
	Present study	1.77–13.35	Topical	Dose-response	4	–	Low-moderate; LD50 focus

a RR is the resistance ratio, calculated as LD50 (or LT50) of field-collected strain/ LD50 (or LT50) of a reference susceptible strain.

b Artificially selected population(s).

c RR >17 is based on the observation that the LD50 of the susceptible strain was 2 ng, and 34.5 ng (10-fold the LD99) failed to kill 50% of the cockroaches.

d One of the strains (Cincy) also was used by Wang et al. (2004) and had an RR = 8.6.

e The same 6 populations were examined by Chai and Lee (2010a) and Ang et al. (2013).

f RR >27.7 is based on the observation that the LD50 of the susceptible strain was 1.3 ng, and 36 ng (10-fold the LD_95_) killed 20–70% of the cockroaches.

**Fig. 5:**
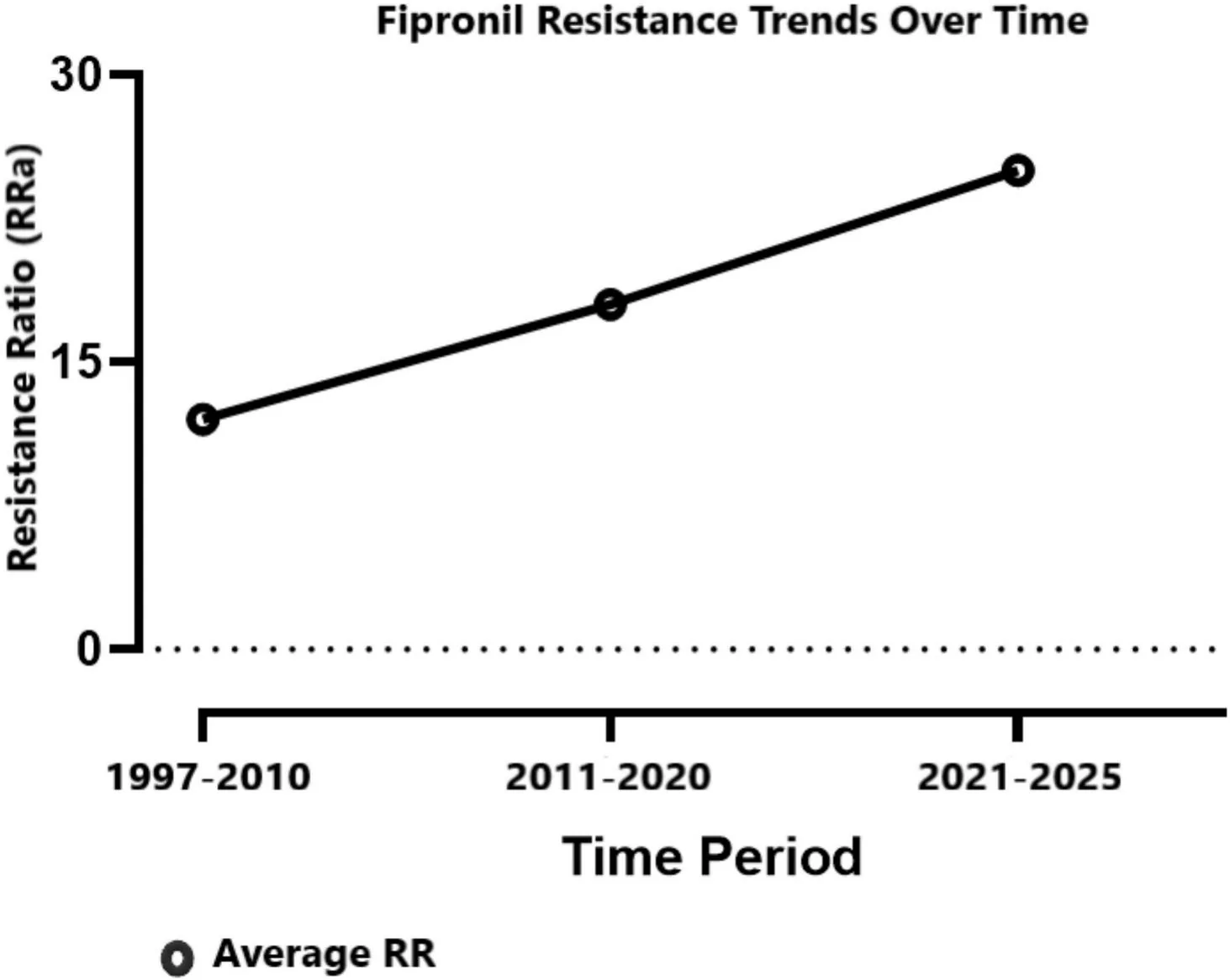
Temporal trends in fipronil resistance development in german cockroach (*Blattella germanica*) populations.

A global review indicates a consistent upward trend in fipronil resistance in *B. germanica* across multiple continents (Table 4). Studies from North America (González-Morales et al., 2022), Asia (R.-Y. Chai and Lee, 2010b; H Nasirian, 2007), and Europe (Kristensen et al., 2005) have documented resistance factors reaching up to 100-fold in some populations. This pattern reflects strong selection pressure imposed by repeated fipronil applications in urban environments, which favors the survival and spread of resistant genotypes (Fig. 5). In Iran, clear geographic variation in fipronil resistance was observed among field populations. Resistance ratios (RR), calculated relative to the susceptible strain (LD₅₀ = 0.68 ppm), ranged from low to moderate in Dormitory and Apartment populations (RR = 1.77–3.36). In these settings, fipronil may still provide effective control when integrated with optimized application strategies and regular resistance monitoring (Kristensen et al., 2005; Scott and Wen, 1997; Valles et al., 1997). In contrast, Fast-food restaurant and Hospital populations exhibited high resistance levels (RR = 12.48–13.35), suggesting that standard fipronil treatments may no longer be reliable in these environments (Gondhalekar and Scharf, 2012; González-Morales et al., 2022; Holbrook et al., 2003; S.-H. Lee et al., 2022). The relatively low probit slope values (0.49–1.07) further indicate substantial heterogeneity in susceptibility within populations, likely reflecting underlying genetic and physiological diversity and the possible coexistence of multiple resistance mechanisms (Abbott, 1925; Finney, 1971; Robertson et al., 2017).

The present study also provides new insights into the molecular basis of resistance by identifying two previously unreported amino acid substitutions in the Rdl gene of *B. germanica*: A299S and A301S. While the well-known A302S mutation was not detected, substitutions at nearby positions (including A296S, A299S, and A301S) have been linked to altered GABA receptor function and insecticide resistance in other insects such as *Anopheles* spp., *Musca domestica*, *Nilaparvata lugens*, and *Drosophila melanogaster* (Garrood et al., 2017; Liu et al., 2020; Ozoe et al., 2015; Remnant et al., 2014). The presence of these variants in a highly conserved region of the GABA-gated chloride channel suggests they may represent novel adaptive changes under insecticide selection pressure.

Haplotype analysis of a 245-bp fragment of the Rdl gene revealed exceptionally high genetic diversity, with 17 unique haplotypes among 21 individuals (Hd = 1.000 ± 0.020; π = 0.0405 ± 0.007), 24 polymorphic sites, and 27 mutations. The haplotype network showed a star-shaped pattern with Hap_18 (accession MW267921.1, USA) at the center, likely representing an ancestral haplotype. Hospital and fast-food restaurant populations displayed greater mutational distances, while the laboratory strain exhibited lower diversity and clustered near the central haplotype. This high level of standing genetic variation is consistent with the known genetic plasticity of urban cockroach populations under recurrent insecticide pressure (Wada-Katsumata and Schal, 2024; Fardisi et al., 2019) and may be maintained by balancing selection or demographic processes (Nei, 1987). Overall, these findings underscore the genetic complexity of fipronil resistance evolution in *B. germanica* and highlight the need for location-specific resistance management. Integrating phenotypic bioassays with molecular surveillance of Rdl variants will be essential for tracking resistance dynamics. Future studies combining functional validation of novel mutations, broader geographic sampling across Iran, and population genetic analyses will help develop predictive tools for resistance emergence. Such integrated approaches are critical for implementing effective Integrated Pest Management (IPM) strategies and preserving the long-term efficacy of fipronil and other insecticides in urban cockroach control.

## 5 Conclusion

This study evaluated the resistance status of *B. germanica* populations to fipronil in densely populated public environments. Given the widespread use of fipronil-based bait formulations, monitoring resistance in field populations is essential to preserve its effectiveness in urban pest management programs. The results demonstrated variation in susceptibility levels among populations, ranging from susceptible to moderately resistant phenotypes. Molecular analysis of randomly selected samples did not identify any previously validated point mutations known to play a major role in target-site resistance to fipronil. Although the A299S and A301S substitutions were detected in two populations, their contribution to resistance remains unclear. Overall, these findings indicate that fipronil continues to be an effective control agent in several environments; however, the observed reduction in susceptibility in some populations highlights the need for continuous resistance surveillance and the implementation of integrated pest management strategies to delay further resistance development.

## Authors contribution

Writing – review & editing, Methodology, Investigation, Conceptualization. Shahin Saeedi: Writing – review & editing, Writing – original draft, Project administration, Investigation, Conceptualization. Hassan Akrami: Writing – review & editing, Validation, Formal analysis. Kourosh Azizi: Writing – review & editing, Validation, Methodology. Azim Paksa: Writing – review & editing, Software, Investigation. Ahmad gholami: Writing – review & editing, Methodology, Investigation. Mitra Boroomand: Writing, Methodology. Sahar Souri Pilangorgi: Data analysis. Ehsan Saki: Methodology. Mozaffar Vahedi: Methodology. Mehdi Miri: Methodology. Aboozar Soltani: Conceptualization, Validation, Software, review & editing, Methodology.

## CRediT authorship contribution statement

**Shahin Saeedi:** Writing – original draft, Methodology, Investigation, Conceptualization. **Hassan Akrami:** Writing – original draft, Validation, Software, Methodology, Formal analysis, Conceptualization. **Kourosh Azizi:** Writing – original draft, Methodology, Investigation, Formal analysis. **Azim Paksa:** Software, Investigation, Formal analysis, Data curation. **Ahmad Gholami:** Visualization, Validation, Methodology, Investigation. **Mitra Boroomand:** Writing – original draft, Methodology, Investigation. **Sahar Souri Pilangorgi:** Formal analysis, Data curation. **Ehsan Saki:** Methodology, Investigation, Data curation. **Mozaffar Vahedi:** Validation, Software, Methodology, Investigation, Conceptualization. **Mehdi Miri:** Writing – original draft, Validation, Software, Methodology, Formal analysis, Conceptualization. **Aboozar Soltani:** Writing – review & editing, Writing – original draft, Validation, Supervision, Resources, Project administration, Methodology, Funding acquisition, Formal analysis, Conceptualization.

## Consent to participate

Not applicable.

## Consent to publish

Not applicable.

## Ethical approval and consent to participate

All study procedures were conducted in compliance with the Declaration of Helsinki and were approved by the Iran National Committee for Ethics in Biomedical Research (Approval ID: IR.SUMS.REC.1402.605).

## Funding statement

This research was supported by the 10.13039/501100004320Vice-chancellor for Research and Technology Affairs of Shiraz University of Medical Sciences (Grant number: 29379), Shiraz, Iran, and this work is based upon research funded by 10.13039/501100003968Iran National Science Foundation (INSF) under project No.4030985.

## Declaration of competing interest

The authors declare that they have no known competing financial interests or personal relationships that could have appeared to influence the work reported in this paper.

## Data Availability

All data supporting the findings of this study can be obtained from the corresponding authors upon reasonable request.
